# Factors Associated with Poor Quality of Sleep in Construction Workers: A Secondary Data Analysis

**DOI:** 10.3390/ijerph18052279

**Published:** 2021-02-25

**Authors:** Youkyung Kim, Sangeun Lee, Jeeyeon Lim, Soyeon Park, Sojeong Seong, Youngshin Cho, Heejung Kim

**Affiliations:** 1 College of Nursing, Yonsei University, Seoul 03722, Korea; dbrud1225@naver.com (Y.K.); olivejeeyeon@yuhs.ac (J.L.); yshin.cho93@gmail.com (Y.C.); 2 College of Nursing, University of Illinois at Chicago, Chicago, IL 60602, USA; slee685@uic.edu; 3 Department of Smart City Engineering, Hanyang University, Seoul 15588, Korea; soysoyeon@hanyang.ac.kr (S.P.); sjsj601@hanyang.ac.kr (S.S.); 4 Mo-Im Kim Nursing Research Institute, Yonsei University, Seoul 03722, Korea

**Keywords:** sleep quality, construction worker, fatigue, depression

## Abstract

This study aimed to explore factors associated with poor quality of sleep in construction workers. This study was cross-sectional, correlational in design and used secondary data from fatigue instrument development study. We analyzed the data from 206 participants aged over 19 years who worked at construction sites for more than 6 months. We used multivariate binary logistic regression to identify the factors associated with poor quality of sleep. We classified the two sleep quality groups based on the Pittsburgh Sleep Quality Index (PSQI) score, and almost 63% of them were classified as the poor quality of sleep group. Based on multivariate binary logistic regression (Cox and Snell R^2^ = 0.317, Nagelkerke R^2^ = 0.429), the poor quality of sleep group tended to sleep for a shorter duration before the working day, and not only showed lower sleep latency and higher levels of daytime dysfunction and discomfort in daily life, but also had more chronic disease, depressive symptoms, and higher physical fatigue. Our study findings support that there are many modifiable factors associated with poor sleep and a high rate of poor quality of sleep occurred in construction workers. Thus, clinicians should consider providing diverse options for applying interventions to ensure better sleep, fatigue management, and depression prevention in construction workers after considering their unique characteristics.

## 1. Introduction

Sleep health is important for the overall health and quality of life of workers. Workers with sleep disorders experience various physical and mental health problems, including abnormal vital signs, mood disorders, poor quality of life, frequent injuries at work, and even early death [1,2,3,4,5]. Specifically, sleep deprivation and irregular sleep patterns result in serious economic losses amounting to more than hundreds of billions of dollars worldwide [6], including in Japan, United States, and Germany. In Korea, the Health Insurance Review and Assessment Service [7] reported that more than 630,000 people were treated in hospitals for sleep-related problems and accounted for almost 51% of the adults aged 20–50 years who are actively engaged in economic activities. Thus, poor quality of sleep affects not only the workers’ individual health, but also the nation’s productivity.

Among the diverse types of sleep parameters, poor quality of sleep is critically important and frequently experienced by construction workers. They are likely to have a shorter duration of sleep at night or reduced sleep efficiency due to the accumulated fatigue [8,9]. These workers tend to have sleep deprivation that leads to increasing daytime sleepiness during working hours and shift work schedule [8,9,10]. Several previous studies have reported that construction workers were likely to experience fatigue, pain, and specific diseases due to sleep disturbances [10,11,12,13]. Consequently, there is ongoing research to identify the relationship among sleep disturbances, occupational environment for shift workers, and health problems in diverse groups of workers [10,14].

It is common for construction workers to perform high-intensity work for extended hours in an unpleasant environment because of the job characteristics. In this study, we aimed to investigate the association of poor quality of sleep with various physical and mental conditions such as fatigue and depression. Fatigue is an important indicator of the overall physical, physiological, and emotional health status [15]. Fatigue at work may be associated with an imbalance between working hours and recovery times [15]. Accumulated fatigue from work increases irregular sleep patterns and reduces self-care capacities important for individual health and wellbeing [8]. Subsequently, the excessive fatigue may activate a vicious cycle of an increasingly unhealthy lifestyle, uncontrolled stress, poorer sleep, and fatigue accumulation [10]. In particular, construction workers with excessive fatigue are likely to have inefficient sleep habits or experience lower sleep efficiency [16]. Thus, it is important to examine both sleep and fatigue simultaneously because sleep may be considered as the process that helps to recover from the excessive fatigue caused by work demands [17].

In addition, sleep problems are closely related to poor mental health. Sleep disturbances are known to be associated with negative psychological symptoms such as depression and anxiety [18]. Oh et al. [19] found that adults with a high risk of insomnia were likely to have a higher risk of anxiety and depression than those without insomnia. Moreover, those with simultaneous anxiety and depression reported poor sleep health pertaining to overall insomnia, quantitative and qualitative sleep, and daytime sleepiness [19]. According to a cohort study conducted in Japan, male workers with insomnia had about seven times higher risk for the onset of depression than those who did not [5]. Moreover, workers with insomnia reported feeling more stressed and depressed than those with a good quality of sleep, regardless of the working schedule [20]. However, limited information is available to develop specific interventions and care policies to ensure the comprehensive health and safety of construction workers. Therefore, it is necessary to explore both physical and psychological factors related to the poor quality of sleep in construction workers.

## 2. Materials and Methods

### 2.1. Study Design and Participants

This study was a secondary data analysis. The primary study was cross-sectional, correlational in design, that evaluated the psychometrics of the Korean version of the Swedish Occupational Fatigue Inventory (SOFI) among 220 construction workers in 2019 [21]. For this secondary data analysis, the baseline data were used rather than a two-week follow-up observation.

For this secondary data analysis, 206 cases were selected among 220 participants in the primary study. The inclusion criteria for this secondary data analysis were: (1) age over 19 years, (2) worked at construction sites for more than 6 months, (3) could understand the purpose of this study and answer the questionnaires, (4) voluntarily agreed to participate, and (5) had Korean nationality. Thus, 14 participants were excluded due to missing information regarding the selected study variables. Various type of construction workers participated in the study. The participants consisted of 32 daily laborers (15.7%), 41 supervisors and site managers (20.1%), and 131 technical workers (64.2%), such as framer, technicians or engineers, etc. Based on statistical power analysis using G*power 3.1 program (Heinrich-Heine-Universitat Dusseldorf, Düsseldorf, Germany), a minimum sample size of 201 was required for a logistic regression model, considering 15 predicted variables with an effect size = 0.5, α = 0.05 (two-tailed), and power = 0.80. Thus, our sample size of 206 participants was deemed sufficient for the data analysis.

### 2.2. Measures

A questionnaire was used consisting of 126 items regarding (1) general characteristics, (2) sleep parameters, and (3) physical and mental health-related factors.

#### 2.2.1. General Characteristics

Sociodemographic characteristics were self-reported in terms of age, sex, marital status, living arrangement, education, and social economic status (SES). For general health-related characteristics, we included body mass index (BMI), smoking, drinking, exercise, and self-rated health. The number of chronic diseases was classified as the absence of chronic diseases, single chronic disease, and multiple chronic diseases, similar to a previous study [3]. Total sleep time (TST) before working days, and TST on off-duty days, which showed actual sleep duration at night for a month, were obtained using the Pittsburgh Sleep Quality Index (PSQI) [22]. In addition, we included occupational characteristics, such as the type of work, working hours per day, and work intensity.

#### 2.2.2. Sleep Parameters

We assessed the sleep parameters using the PSQI translated to Korean, which was offered by the Mapi Research Trust. The PSQI Index consists of subjective sleep quality, latency, duration, habitual sleep efficiency, disturbance, use of sleep medication, and daytime dysfunction. Total scores of global PSQI range from 0 to 21, with higher scores indicating a poor quality of sleep. For this study, the subjective sleep quality groups were classified according to the PSQI score of 5 because some researchers insisted the PSQI cutoff score should be considered of working condition [23]. Thus, we adopted the PSQI cutoff score of 5, which was tested in Korean workers [24]. The good quality of sleep group had PSQI ≤ 5, while the poor quality of sleep group had PSQI > 5 [22,23,24]. The Cronbach’s α coefficient was reported to be 0.83 in the original study [22], 0.80 in a study on Korean occupational drivers [25], and 0.65 in our study.

#### 2.2.3. Physical and Mental Health-Related Factors

We assessed multidimensional factors of physical and mental health using long-term and occupational fatigue and depression.

The Multidimensional Fatigue Scale (MFS) was developed by Schwartz et al. [26] and was translated into Korean by Chang et al. [27]. The Korean version of MFS consists of 19 items (eight items on global fatigue, six items on impact on daily functioning, and five items on situation specific fatigue) with a seven-point Likert-type scale (from 1 = completely disagree to 7 = completely agree). Total scores of the instrument range from 19 to 133, with higher scores indicating higher levels of fatigue. The Cronbach’s α coefficients were 0.88 overall (0.85 for global fatigue, 0.79 for impact on daily functioning, and 0.66 for situation specific fatigue) in the original study [26] and 0.94 (0.90 for global fatigue, 0.89 for impact on daily functioning, and 0.77 for situation specific fatigue) in this study.

The SOFI was developed by Åhsberg et al. [28] and was translated into Korean by our research team. The instrument consists of 20 items with a seven-point Likert scale (from 0 = not at all to 6 = to a very high degree). Total scores of the instrument range from 0 to 120, with higher scores indicating higher levels of momentary fatigue. The Cronbach’s α coefficients were 0.80 in the original study [28] and 0.96 in this study.

We also assessed fatigue using the Subjective Scale of Fatigue (SSF) developed by the Japan Industrial Hygiene Association Industrial Fatigue Research Committee [29], translated into Korean. This instrument consists of 30 items (10 items on physical fatigue, 10 items on mental fatigue, and 10 items on neurosensory fatigue) with a four-point Likert scale (from 1 = never to 4 = always). Total scores of the instrument range from 30 to 120, with higher scores indicating more severe symptoms due to fatigue. The Cronbach’s α coefficients were reported as 0.80 in the original study (Japan Industrial Hygiene Association Industrial Fatigue Research Committee) [29] and 0.95 in the present study (0.90 for physical fatigue, 0.92 for mental fatigue, and 0.86 for neurosensory fatigue).

The depressive symptoms were assessed using the 10-item short form of the Center for Epidemiologic Studies Depression Scale (CESD-10). The original 20-item of CESD was translated into Korean [30]; we adopted the short version evaluated by Andresen et al. [31]. The instrument consists of 10 items with a four-point Likert scale (from 0 = none of the time to 3 = most of the time). Total scores of the instrument range from 0 to 30, with higher scores indicating more depressive symptoms. The Cronbach’s α coefficient was reportedly 0.81 in a study on general adult drivers [32] and 0.78 in our study.

### 2.3. Analysis

Descriptive statistics were presented as frequencies (percentages) or means (standard deviations [SD]). Independent t-tests (or Mann Whitney U test) and chi-squared statistics were used to compare the two groups. After identifying the statistically significant factors based on univariate analyses, multivariate binary logistic regression was performed to identify the factors associated with the poor quality of sleep group when compared to the good quality of sleep group. Statistical assumptions including multicollinearity among independent variables were checked. There was no multicollinearity between the variables; the variance inflation factor (VIF) of the independent variables ranged from 1.12 to 3.72 and the minimum tolerance was 0.27. Data analysis was conducted with SPSS 25.0 (IBM Corp., Armonk, NY, USA), and the significance level was set at α = 0.05.

### 2.4. Ethical Consideration

For this secondary data analysis, we obtained ethical approval from the affiliated Institutional Review Board (IRB No. Y-2020-0072). All data were de-identified to protect the confidentiality of participants.

## 3. Results

### 3.1. Differences in the General Characteristics between the Two Sleep Quality Groups

The study participants were classified into two groups, with 77 participants in the good quality of sleep group (37.4%) and 129 in the poor quality of sleep group (62.6%). The mean age of the participants was 47.56 ± 13.17 years, with almost 85% of the participants younger than 60. The majority of them were men (85.0%), married (73.8%), and living with others (90.8%). Almost half of the participants were educated up to the diploma level or above (49.0%) and had moderate SES (43.2%). The average BMI was 24.16 ± 2.86 kg/m^2^; 35.5% participants had normal (18.5–22.9) BMI, 30.6% were overweight (23.0–24.9), and 33.9% were obese (≥25.0). Furthermore, more than half were current smokers (52.4%), drank alcohol (73.8%), and did regular workouts (57.4%). Almost one-third (31.6%, 65/206) of the participants were diagnosed with chronic diseases, and the most common diseases were hypertension (18.9%), diabetes (5.3%), and gastrointestinal disease (4.9%). Almost 60% participants identified themselves as having a moderate health status. The majority of participants were regular workers with a 9 am to 5 pm schedule (80%); more than half of them worked over 8 h per day (67.5%) and did high intensity work (51.0%). There were significant differences identified between the two groups in terms of marital status, SES, the number of chronic diseases, self-rated health, and work intensity (see details in Table 1).

### 3.2. Differences in the Sleep Parameters between the Two Sleep Quality Groups

The differences in the sleep parameters between the two groups are shown in Table 2. In general, there were significant differences in the sleep parameters except for the TST on off-duty days between the two groups. That is, the poor quality of sleep group had a worse subjective sleep quality, longer sleep latency, shorter sleep duration, and worse habitual sleep efficiency compared to the good quality of sleep group. They also reported more sleep disturbance, more daytime dysfunction, and higher use of sleep medications in the past month compared to the good quality of sleep group (all *p* values < 0.01). The poor quality of sleep group reported a shorter TST before working days along with a high score of PSQI compared to the good quality of sleep group (*p* < 0.001). However, the mean TST on off-duty days of the participants was 8.35 ± 2.14 h and there was no significant difference between the two groups.

### 3.3. Differences in the Physical and Mental Health-Related Characteristics between the Two Sleep Quality Groups

Table 3 shows the differences in the physical and mental health-related characteristics between the two sleep quality groups. There were significant differences in all health-related factors between the two groups. Participants in the poor quality of sleep group reported significantly higher CESD-10 (*p* < 0.001), MFS (*p* < 0.001), SOFI (*p* < 0.001), and SSF (*p =* 0.001) scores than the good quality of sleep group. In summary, those with a poor quality of sleep were more likely to be depressed and experienced higher levels of fatigue than those with a good quality of sleep.

### 3.4. Multivariate Binary Logistic Regression Analysis

Multivariate binary logistic regression analysis was undertaken to identify the significant factors associated with the two groups (Table 4). The model significantly explained the classification between the good and poor quality of sleep groups (Cox and Snell R^2^ = 0.317, Nagelkerke R^2^ = 0.429). Overall, the significant factors associated with the quality of sleep were the number of chronic diseases (odds ratio [OR] = 6.97, confidence intervals [CI] 2.25, 21.57 for one chronic disease), CESD-10 (OR = 1.15, CI = 1.02, 1.30), and physical fatigue of SSF (OR = 1.20, CI = 1.02, 1.42). Compared to those with a good quality of sleep, those with a poor quality of sleep were more likely to have a chronic disease, more depressive symptoms, and higher physical fatigue.

## 4. Discussion

The present study investigated the association of the quality of sleep with physical and mental health factors in construction workers. Our study findings suggest that the majority of construction workers perceived the poor quality of sleep both quantitatively and qualitatively. In addition, construction workers with a poor quality of sleep reported higher levels of depression, long-term fatigue, and momentary fatigue. Significant factors associated with the quality of sleep were the number of chronic diseases, depression, and physical fatigue.

Two-thirds of the construction workers were classified as ‘the poor quality of sleep group’ and the two groups presented different profiles with regard to both quantitative and qualitative sleep; the proportion of participants (63%) in the poor sleep group in this study was relatively high. According to previous studies, the poor quality of sleep group comprised 29.1% of electronic workers, 37.4% of public workers, and 54% of police officers [33,34,35]. In Japan, a study found a strong association between the working hours and sleep disorders, including insomnia and daytime sleepiness [36]. In addition, a study on shift workers showed that working hours artificially changed the biorhythm, causing a reduction in the quality of sleep [18]. Our study revealed more details about the specific sleep parameters related to the poor quality of sleep in construction workers. Compared to the good quality of sleep group, the poor quality of sleep group generally tended to sleep for a shorter duration before the working day, and showed lower sleep latency as well as higher levels of daytime dysfunction and discomfort in daily life. The negative sleep pattern may increase incomplete recovery at the beginning of a new work shift [17]. Moreover, the poor quality of sleep group reported bad sleep habits, such as the frequent use of sleep medications. In general, some studies found evidence to support the effect of non-pharmacological interventions to improve the quality of sleep such as exercise [37,38], cognitive and behavioral treatments [39], and utilizing mobile health (mHealth) intervention [40]. However, occupational and environmental characteristics of construction workers should be considered when implementing specific interventions for sleep management due to different sleep patterns.

Physical fatigue is a significant parameter related to poor sleep. Specifically, the poor quality of sleep group in our study reported more physical fatigue than mental or neurosensory fatigue compared to the good quality of sleep group. This finding is very important to develop multidimensional assessment because construction workers who felt tired or exhausted were more likely to report physical difficulty rather than emotional or cognitive dysfunction [41]. Thus, detection of physical symptoms of fatigue could be an early assessment tool or used in the initial steps of the intervention to prevent other aspects of fatigue and negative consequences. However, caution is required when using a new device with physical feature changes. For example, a previous study developed a new device, an inverted foot lever or drill press, to reduce physical strain among construction workers. However, the construction workers reported feeling unfamiliar with the use of the new device and were afraid of the risk of injuries due to the difficulty in using the device [42]. Thus, the occupational health care providers should think of the pros and cons of implementing physical interventions for better health, safety, and productivity of specific workers.

In our study, long-term and momentary fatigue differed between the two groups simultaneously. Although substantial research has been conducted among occupational groups for better understanding of long-term fatigue and poor sleep [43], very few studies have explored both types of fatigue in relation to sleep health [17,44] similar to our study. Andrei et al. [17] found that both accumulated and temporary fatigue were simultaneously associated with the poor quality of sleep in maritime workers. Their study emphasized that fatigue should be distinguished into acute and sustained fatigue because it not only occurs over extended time periods, but is also an instant response to the stressors in a specific situation [17]. Based on the job demand-resource model, repetitive excess of energy expenditure or preservation results in prolonged recovery, accumulated exhaustion, and long-term fatigue [17]. In our study, the poor quality of sleep may be considered as an insufficient or ineffective recovery. However, our findings cannot confirm the mediation model of poor sleep between acute and chronic fatigue due to the cross-sectional design. Therefore, more longitudinal research is required to assess both short-term and long-term fatigue, which influence sleep health over time.

Depressive mood is also associated with the poor quality of sleep in construction workers. Consistent with previous studies [19,45], we confirmed that participants with a poor quality of sleep were more likely to have depressive symptoms than those with a good quality of sleep. For example, Oh et al. [19] reported that 47.6% of people with a high risk of insomnia had comorbid psychiatric problems, such as anxiety or depression. These studies also found that participants with both anxiety and depression were more likely to have difficulties in maintaining sleep than people without anxiety or depression, or with anxiety only. However, another study reported that the likelihood of newly developed depression in the future could increase with the severity of insomnia [5]. Nishitani et al. [5] identified that people with at least minor insomnia symptoms (the Athens Insomnia Scale (AIS) score ≥ 1) at baseline had about 7.1 times more risk of depression than people with no insomnia symptoms at all. Due to the cross-sectional design of our study, we cannot confirm a specific relationship between depression and sleep in terms of the predisposing factors, comorbid conditions, or the symptoms. However, our study findings emphasize that a multicomponent approach including both depression and sleep could be beneficial for alleviating the two conditions simultaneously. Thus, it is recommended that evidence-based interventions should be provided for construction workers through diverse modes of interventions [46,47], for example of the Internet-based cognitive behavioral therapy for insomnia (ICBT-i). Thus, the occupational healthcare providers should consider providing diverse options for applying multicomponent interventions in order to ensure better sleep and depression prevention in construction workers.

Managing chronic diseases is important in the poor sleep quality group. In general, poor sleep is associated with not only mental health problems, but also physical diseases. For example, poor quantity and quality of sleep increase the likelihood of experiencing problems with concentration, fatigue, and depression [48]. In addition, sleep deprivation is known as a risk factor for cardiovascular disease, respiratory disease, and diabetes [48]. Prihatiningsih [49] reported that workers with an abnormal sleep duration (<6 hours, or >8 hours) tend to have a higher average systolic blood pressure, which can lead to hypertension, compared to workers with a normal sleep duration. A short sleep duration (<7 hours) is also associated with physical and mental diseases including depression, impaired immune function, and cardiovascular disease [50]. Thus, policy makers should consider applying a quantitative and qualitative sleep management policy for improving the health of construction workers in order to prevent chronic diseases. Annual and regular checkups should be available for early identification of health problems and integrated management of body and mind.

### Limitations

This study has several limitations due to the study design and methodology. First, there is the possibility of a measurement error due to the use of only self-reported data. Previous study has reported that physical workload and perceived fatigue (SOFI) are significantly related [51] and objective measure of sleep is also important [50]. Therefore, further studies should use actigraphic devices to measure objective sleep and fatigue to overcome the limitations of self-reported data. Second, we did not consider the characteristics of the construction site or the type of work. Future studies should reflect the variables in the working environment or the specific tasks performed in terms of the inclement weather, use of heavy or dangerous equipment, and different work site changes along with demanding and extended work hours. Third, it may be difficult to generalize our results because of the cross-sectional design, small sample size, convenience sampling and participants are composed only of Korean men. Consequently, we recommend the future studies with larger samples recruited from multiple working sites, specifically purposive sampling including women and foreign workers.

## 5. Conclusions

Our study findings support the importance to identify the factors associated with the poor quality of sleep in construction workers to promote better sleep health. This secondary data analysis revealed that the majority of construction workers perceived poor quality of sleep. Specifically, there are many modifiable factors associated with poor sleep, such as long-term and momentary fatigue specifically related to physical aspects, depressive mood related to mental health, and chronic diseases. It is necessary to develop comprehensive programs to integrate all physical, mental, and sleep health components at the same time. Although our study tried to differentiate time-dependent fatigue, it is also necessary to consider the recovery process within the specific work demands among construction workers over time.

## Figures and Tables

**Table 1:** Differences of general characteristics between the two sleep quality groups (*N* = 206).

Variables	Categories	Total *N* (%)	Good Quality of Sleep Group *n* (%)	Poor Quality of Sleep Group *n* (%)	*χ*^2^*(p*)
Age (years) *	<60	163 (84.5)	61 (83.6)	102 (85.0)	0.072 (0.789)
	≥60	30 (15.5)	12 (16.4)	18 (15.0)	
Sex	Male	175 (85.0)	66 (85.7)	109 (84.5)	0.056 (0.813)
	Female	31 (15.0)	11 (14.3)	20 (15.5)	
Marital status	Married	152 (73.8)	63 (81.8)	89 (69.0)	4.101 (0.043)
	Not married	54 (26.2)	14 (18.2)	40 (31.0)	
Living arrangement	Living alone	19 (9.2)	6 (7.8)	13 (10.1)	0.301 (0.583)
	Living with others	187 (90.8)	71 (92.2)	116 (89.9)	
Education	Up to middle school	26 (12.6)	7 (9.1)	19 (14.7)	1.676 (0.433)
	High school	79 (38.4)	29 (37.7)	50 (38.8)	
	Diploma or above	101 (49.0)	41 (53.2)	60 (46.5)	
Social economic status	High	30 (14.6)	18 (23.4)	12 (9.3)	9.361 (0.009)
	Moderate	89 (43.2)	34 (44.1)	55 (42.6)	
	Low	87 (42.2)	25 (32.5)	62 (48.1)	
BMI (kg/m^2^) *	Normal (18.5–22.9)	66 (35.5)	25 (35.7)	41 (35.3)	1.874 (0.392)
	Overweight (23.0–24.9)	57 (30.6)	25 (35.7)	32 (27.6)	
	Obese (≥25)	63 (33.9)	20 (28.6)	43 (37.1)	
Smoking	Current smoker	108 (52.4)	43 (55.8)	65 (50.4)	0.576 (0.448)
	Not current smoker	98 (47.6)	34 (44.2)	64 (49.6)	
Drinking	Current drinker	152 (73.8)	52 (67.5)	100 (77.5)	2.486 (0.115)
	Not current drinker	54 (26.2)	25 (32.5)	29 (22.5)	
Regular exercise *	Yes	117 (57.4)	46 (61.3)	71 (55.0)	0.768 (0.381)
	No	87 (42.6)	29 (38.7)	58 (45.0)	
The number of chronic diseases	0	141 (68.4)	67 (87.0)	74 (57.4)	19.661 (<0.001)
	1	50 (24.3)	8 (10.4)	42 (32.5)	
	2 or more	15 (7.3)	2 (2.6)	13 (10.1)	
Self-rated health	Good	34 (16.5)	24 (31.2)	10 (7.8)	20.830 (<0.001)
	Moderate	121 (58.7)	41 (53.2)	80 (62.0)	
	Poor	51 (24.8)	12 (15.6)	39 (30.2)	
Work type	Regular work	176 (85.4)	66 (85.7)	110 (85.3)	0.008 (0.931)
	Irregular work	30 (14.6)	11 (14.3)	19 (14.7)	
Working hours per day	≤8	67 (32.5)	29 (37.7)	38 (29.5)	1.479 (0.224)
	>8	139 (67.5)	48 (62.3)	91 (70.5)	
Work intensity	Hard	105 (51.0)	30 (39.0)	75 (58.1)	7.097 (0.008)
	Moderate or not hard	101 (49.0)	47 (61.0)	54 (41.9)	

Note. BMI; Body mass index, * Missing data were excluded from the analyses.

**Table 2:** Differences of sleep parameter between the two sleep quality groups (*N* = 206).

Variables	Range	Total *N* (%) or M ± SD	Good Quality of Sleep Group *n* (%) or M ± SD	Poor Quality of Sleep Group *n* (%) or M ± SD	*p*
TST before working days (hour) *		6.72 ± 1.07	7.08 ± 1.01	6.51 ± 1.06	<0.001
TST at off-day (hour) ^a,^*		8.35 ± 2.14	8.28 ± 2.16	8.40 ± 2.13	0.877
Total PSQI score ^a,^*	0–21	6.37 ± 2.83	3.75 ± 1.17	8.27 ± 2.05	<0.001
Subjective sleep quality ^a,^*	0–3	1.16 ± 0.67	0.82 ± 0.51	1.37 ± 0.68	<0.001
Sleep latency (minute)	≤15	28 (13.6)	18 (23.4)	10 (7.8)	0.002
	>15	178 (86.4)	59 (76.6)	119 (92.2)	
Sleep duration (hour)	>7	41 (19.9)	28 (36.4)	13 (10.1)	<0.001
	≤7	165 (80.1)	49 (63.6)	116 (89.9)	
Habitual sleep efficiency (%)	≥85	162 (78.6)	76 (98.7)	86 (66.7)	<0.001
	<85	44 (21.4)	1 (1.3)	43 (33.3)	
Sleep disturbance ^a,^*	0–27	6.23 ± 4.07	4.06 ± 2.90	7.60 ± 4.11	<0.001
Use of sleeping medication in the past month	Yes	19 (9.2)	1 (1.3)	18 (14.0)	0.002
	No	187 (90.8)	76 (98.7)	111 (86.0)	
Daytime dysfunction ^a,^*	0–3	1.23 ± 0.81	0.69 ± 0.67	1.55 ± 0.70	<0.001

Note. TST: Total sleep time; PSQI: Pittsburgh Sleep Quality Index; * Missing data were excluded from the analyses; ^a^ Data were tested by Mann-Whitney U test.

**Table 3:** Differences of physical and mental health related characteristics between the two sleep quality groups (*N* = 206).

Variables	Subcategories	Total (M ± SD)	Good Quality of Sleep Group (M ± SD)	Poor Quality of Sleep Group (M ± SD)	*p*
		*n* = 206	*n* = 77	*n* = 129	
CESD-10 *		6.66 ± 4.45	4.88 ± 3.84	7.76 ± 4.45	<0.001
MFS *	Total	56.77 ± 21.02	50.59 ± 23.89	60.46 ± 18.23	<0.001
	Global fatigue	23.53 ± 10.02	21.19 ± 11.09	24.91 ± 9.11	0.006
	Impact on daily functioning	19.11 ± 7.76	16.64 ± 8.62	20.57 ± 6.83	<0.001
	Situation specific fatigue	14.28 ± 5.93	12.90 ± 6.16	15.12 ± 5.64	0.006
SOFI *	Total	42.50 ± 24.83	32.29 ± 23.43	49.04 ± 23.54	<0.001
	Physical exertion	6.00 ± 4.99	4.29 ± 4.90	7.02 ± 4.78	<0.001
	Lack of motivation	8.63 ± 5.36	6.15 ± 4.77	10.06 ± 5.17	<0.001
	Lack of energy	9.53 ± 5.75	7.28 ± 5.71	10.91 ± 5.34	<0.001
	Physical discomfort	8.13 ± 5.68	5.56 ± 5.08	9.70 ± 5.47	<0.001
	Sleepiness	10.48 ± 5.94	8.36 ± 6.42	11.71 ± 5.28	<0.001
SSF *	Total	56.70 ± 17.98	54.90 ± 20.40	62.72 ± 15.63	0.001
	Physical fatigue	21.11 ± 6.31	18.83 ± 6.84	22.44 ± 5.58	<0.001
	Mental fatigue	19.84 ± 6.50	18.14 ± 7.14	20.84 ± 5.89	0.001
	Neurosensory fatigue	18.89 ± 6.98	17.03 ± 7.63	20.03 ± 6.32	<0.001

Note. CESD-10: The form of the Center for Epidemiologic Studies Depression scale; MFS: Multidimensional Fatigue Scale; SOFI: Swedish Occupational Fatigue Inventory; SSF: Subjective Scale of Fatigue; * Missing data were excluded from the analyses; All data were tested by Mann-Whitney U test.

**Table 4:** Multivariate binomial logistic regression analysis with the two sleep quality groups (*N* = 206).

Variables	Categories	B	S.E.	*p* Value	Odd Ratio	95% CI
Marital group (ref. married)	Not married	0.54	0.48	0.259	1.71	0.67–4.35
Social economic status (ref. moderate)	High	−0.94	0.67	0.158	0.39	0.11–1.44
	Low	0.29	0.46	0.522	1.34	0.55–3.26
The number of chronic diseases (ref. 0)	1	1.94	0.58	0.001	6.97	2.25–21.57
	2 or more	1.75	0.87	0.056	9.19	0.94–89.50
Self-rated health (ref. good)	Moderate	1.00	0.58	0.083	2.71	0.88–8.37
	Poor	−0.21	0.78	0.784	0.81	0.18–3.72
Work intensity (ref. hard)	Moderate or not hard	0.01	0.47	0.985	1.01	0.41–2.51
CESD-10		0.14	0.06	0.024	1.15	1.02–1.30
MFS	Global fatigue	−0.00	0.03	0.891	1.00	0.94–1.06
	Impact on daily functioning	0.04	0.05	0.443	1.04	0.95–1.13
	Situation specific fatigue	−0.05	0.06	0.386	0.95	0.85–1.06
SSF	Physical fatigue	0.18	0.09	0.033	1.20	1.02–1.42
	Mental fatigue	−0.08	0.06	0.211	0.92	0.82–1.05
	Neurosensory fatigue	−0.04	0.06	0.542	0.97	0.86–1.08
SOFI		0.02	0.01	0.160	10.02	0.99–1.04
	Cox and Snell R^2^ = 0.317					
	Nagelkerke R^2^ = 0.429					

Note. Logistic regression with complex sample was conducted with the two sleep quality groups as a referent; ref.: reference of each independent variable group; CESD-10: The short form of the Center for Epidemiologic Studies Depression scale; MFS: Multidimensional Fatigue Scale; SSF: Subjective Scale of Fatigue; SOFI: Swedish Occupational Fatigue Inventory.

## Data Availability

The data presented in this study are available on request from the corresponding author. The data are not publicly available due to protection of subjects’ privacy and confidentiality.
